# Research progress on the relationship between bile acid metabolism and type 2 diabetes mellitus

**DOI:** 10.1186/s13098-023-01207-6

**Published:** 2023-11-17

**Authors:** Yisen Hou, Xinzhe Zhai, Xiaotao Wang, Yi Wu, Heyue Wang, Yaxin Qin, Jianli Han, Yong Meng

**Affiliations:** 1 Department of Oncology Surgery, Xi’an No.3 Hospital, The Affiliated Hospital of Northwest University, Xi’an, 710018 Shanxi People’s Republic of China; 2 Department of General Surgery, Shanxi Bethune Hospital, Shanxi Academy of Medical Sciences, Third Hospital of Shanxi Medical University, Taiyuan, 030032 Shanxi People’s Republic of China

**Keywords:** Bile acid, Type 2 diabetes mellitus, Farnesoid X receptor, Takeda G-protein coupled receptor 5, Fibroblast growth factor 19

## Abstract

Bile acids, which are steroid molecules originating from cholesterol and synthesized in the liver, play a pivotal role in regulating glucose metabolism and maintaining energy balance. Upon release into the intestine alongside bile, they activate various nuclear and membrane receptors, influencing crucial processes. These bile acids have emerged as significant contributors to managing type 2 diabetes mellitus, a complex clinical syndrome primarily driven by insulin resistance. Bile acids substantially lower blood glucose levels through multiple pathways: BA-FXR-SHP, BA-FXR-FGFR15/19, BA-TGR5-GLP-1, and BA-TGR5-cAMP. They also impact blood glucose regulation by influencing intestinal flora, endoplasmic reticulum stress, and bitter taste receptors. Collectively, these regulatory mechanisms enhance insulin sensitivity, stimulate insulin secretion, and boost energy expenditure. This review aims to comprehensively explore the interplay between bile acid metabolism and T2DM, focusing on primary regulatory pathways. By examining the latest advancements in our understanding of these interactions, we aim to illuminate potential therapeutic strategies and identify areas for future research. Additionally, this review critically assesses current research limitations to contribute to the effective management of T2DM.

## Introduction

Type 2 diabetes mellitus (T2DM) is a complex clinical syndrome characterized by disrupted glucose metabolism, due to the interplay of genetic and environmental factors. The proportion of T2DM in patients with diabetes is estimated to be 90%-95% [1–3]. The escalating burden of T2DM poses significant challenges to public health, with nearly 500 million affected individuals worldwide, and projections indicating a substantial increase in the future [4]. Insulin resistance and pancreatic β-cell dysfunction lie at the core of T2DM pathogenesis. Insulin resistance refers to the impaired response of target tissues, including adipose, liver, and skeletal muscle, to the actions of insulin, leading to diminished glucose uptake. Simultaneously, β-cell dysfunction results in inadequate insulin secretion to compensate for insulin resistance, further contributing to hyperglycemia [1–3].

The etiology of T2DM involves intricate interactions between genetic and environmental factors. Genetic susceptibility, characterized by polymorphisms in genes related to insulin secretion, insulin action, and β-cell function, contributes to an increased predisposition to the disease. Environmental factors, such as sedentary lifestyles, unhealthy dietary patterns, and obesity, interact synergistically with genetic factors, influencing the development of T2DM [2, 5]. Obesity plays a pivotal role in the pathogenesis of T2DM. Excessive adipose tissue, especially in visceral depots, induces a state of chronic low-grade inflammation and dysregulated secretion of adipokines, impairing insulin signaling pathways and exacerbating insulin resistance. Adipose tissue dysfunction further leads to the release of free fatty acids and adipokines, which contributes to peripheral tissue insulin resistance [6, 7].

Bile acids (BAs), synthesized from cholesterol in the liver and excreted into the bile, extend beyond their traditional role in fat absorption and cholesterol homeostasis. Emerging evidence suggests crucial involvement in metabolic regulation. Acting as signaling molecules, BAs activate various receptors, including the farnesoid X receptor (FXR) and the G-protein-coupled bile acid receptor 5 (TGR5), in the liver and peripheral tissues. Activation of these receptors exerts modulatory effects on glucose and lipid metabolism, enhances insulin sensitivity, and maintains energy homeostasis [8–10].

The intricate relationship between BAs and the pathogenesis of T2DM has gained significant attention in recent research [11–13]. Targeting the bile acid signaling pathways has emerged as a potential therapeutic strategy for T2DM management. Elucidating the precise mechanisms underlying the metabolic effects of BAs and exploring their therapeutic implications hold promise for innovative interventions in T2DM treatment.

Given the rising global prevalence of T2DM and the expanding recognition of BAs' role in metabolic regulation, comprehensive investigations into the fundamental mechanisms of T2DM pathogenesis and the therapeutic potential of bile acid signaling pathways are of utmost importance. Such research endeavors provide a platform for novel insights into T2DM management, fostering the development of innovative therapeutic approaches that hold the potential to enhance patient outcomes in this prevalent metabolic disorder.

## Synthesis and recovery of Bile acid

**Fig. 1:**
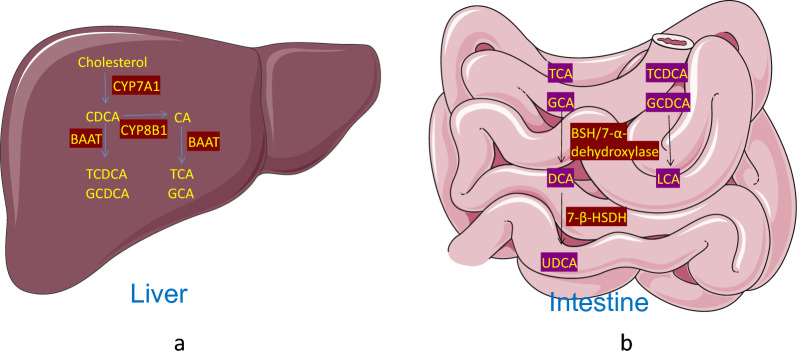
The synthesis of primary and secondary bile acids. **a** CDCA and CA are predominantly synthesized through the classical pathway in the hepatic endoplasmic reticulum, contributing to more than 90% of total bile acid synthesis under normal physiological conditions. This synthesis process is regulated by CYP7A1, and CDCA is produced in the absence of CYP8B1. Subsequently, these primary bile acids are converted into conjugated forms, primarily glycine or taurine-conjugated (in a 3:1 ratio in humans), with the assistance of BAAT. **b** Within the intestine, BSH plays a predominant role in deconjugating bile acids (TCA, GCA, TCDCA, and GCDCA), converting them back into unconjugated forms. Subsequently, 7-α-dehydroxylase enzymes catalyze the conversion of these unconjugated bile acids into DCA and LCA. Additionally, a minor fraction of deoxycholic acid can be further converted into UDCA by intestinal bacteria's 7-β-hydroxysteroid Dehydrogenase enzymes

**Fig. 2:**
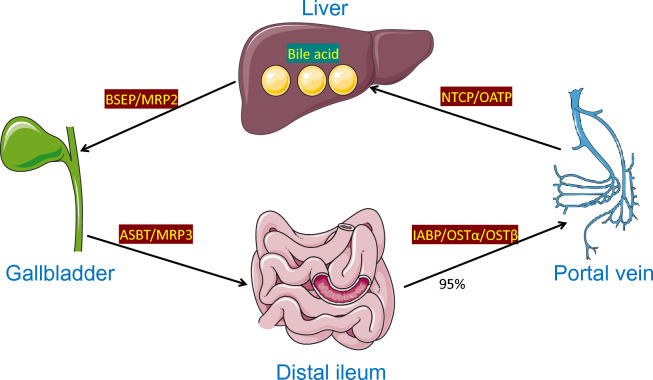
the enterohepatic circulation of BAs. Initially, primary BAs synthesized in the liver are stored in the gallbladder with the assistance of BSEP and MRP2. In the distal ileum, roughly 95% of intestinal BAs are actively reabsorbed through ASBT and MRP3. Bile acids, facilitated by IABP, traverse intestinal cells and then enter the portal circulation via OST α/OST β. In the hepatocyte basement membrane, NTCP and OATP transport the absorbed BAs to the hepatic sinusoid through active transport mechanisms

Bile acids are byproducts of cholesterol primarily synthesized in the liver, with about 0.4–0.6 g of the daily synthesized 1–1.5 g cholesterol converted into bile acids. This synthesis occurs through two pathways: the classical pathway and the alternative pathway. The classical pathway, responsible for over 90% of bile acid synthesis, occurs in the hepatic endoplasmic reticulum and is mediated by cholesterol 7-α-hydroxylase (CYP7A1) [3, 13, 14]. In its absence, chenodeoxy cholic acid (CDCA) is produced. The acidic pathway is mediated by cholesterol 27-α-hydroxylase (CYP8B1), primarily in peripheral tissues and in macrophages [12, 14–16]. (Fig. 1a). These primary bile acids are conjugated to glycine or taurine (approximately 3:1 in humans) by Bile acid CoA: amino acid N-acyltransferase (BAAT). These conjugated bile acids are then absorbed by hepatocytes through bile salt export pump (BSEP) and multidrug resistance-associated protein 2 (MRP2). They are stored in the gallbladder, and later released into the intestine during feeding [17–19]. About 95% of intestinal BA is actively reabsorbed by intestinal cells from the distal ileum through apical sodium-dependent transporter (ASBT) or multidrug resistance-associated protein 3 (MRP3). Bile acids pass through intestinal epithelial cells, facilitated by ileal bile acid binding protein (IABP), to reach the basolateral membrane, and finally pass through the heterodimer of organic solute transporter α and β (OST α / OST β) into the portal vein circulation [12, 13, 17, 20]. Sodium-dependent taurocholate cotransporting polypeptide (NTCP), located in the hepatocyte basement membrane, is responsible for facilitating sodium-dependent binding. Meanwhile, organic anion transporter (OATP), also found in the hepatocyte basement membrane, handles the uptake of unconjugated BAs. Afterward, active transporters within the hepatic sinusoid membrane of hepatocytes efficiently clear these BAs. These newly dissociated BAs are returned to the hepatocytes with newly formed BAs and then secreted into the bile duct, a process known as enterohepatic circulation [13, 14, 18] (Fig. 2). Approximately 5% of bile salts escape this circulation and are transformed by intestinal microflora. Bile salt hydrolase enzymes (BSH) deconjugate bile salts, and 7-α-dehydroxylase enzymes convert unconjugated bile acids into deoxycholic acid (DCA) and lithocholic acid (LCA). Some deoxycholic acid can be further converted into ursodeoxycholic acid (UDCA) [16, 21, 22]. (Fig. 1b). Finally, the human bile acid pool primarily consists of CA, CDCA, and DCA in a ratio of 40:40:20 [3, 16]. While in mice, it is mainly composed of Taurocholic acid (TCA), T-β-muricholic acid, and T-α-muricholic acid in a ratio of 60:40 (TCA: TMCA) [12, 14].

The discrepancy in bile acid composition between mice and humans can primarily be attributed to the presence of a species-specific sterol 6-β-hydroxylase enzyme, Cyp2c70, exclusively found in mice. Notably, this enzyme is absent in humans, limiting the conversion of CDCA to α-muricholic acid (α-MCA) in the human hepatic system [23]. In addition to the enzyme disparity, other factors such as dietary structure, intestinal flora, and genetic variations may contribute to the observed differences. The interplay of BSH activity and ileal bile acid binding protein further influences bile acid metabolism, ultimately shaping the distinct bile acid profiles between species [24, 25]. Therefore, when extrapolating findings based on animal models, the applicability of changes in bile acid metabolism in these models to the human system must be carefully considered, especially in the context of T2DM-like diseases. Further studies are needed to fully understand the impact of species-specific bile acid metabolism changes and their relevance to human physiology.

## Bile acid and type 2 diabetes mellitus

T2DM is a common disease characterized by abnormal elevation of blood glucose. As mentioned earlier, insulin resistance is considered to be the main pathogenesis of T2DM [2, 5]. Insulin resistance in insulin-sensitive tissues can lead to increased insulin secretion by pancreatic β-cells, but the final result is that β-cells cannot meet the increased demand for insulin. Furthermore, oxidative stress, autophagy and apoptosis of β cells change the function of pancreatic islets [6, 7]. In animal experiments and clinical studies, diabetes and insulin resistance are associated with the increase of 12 α-hydroxylated BA (CA/GCA/DCA) / non-12 α-hydroxylated BA [26–30]. The reason for this may be that abnormally elevated levels of glucose and insulin can promote histone acetylation of CYP7A1 chromatin, thus stimulating the synthesis of CYP7A1 and resulting in an increase in 12 α-hydroxylation BA production. Interestingly, alterations in bile acid composition can, in turn, improve blood glucose disorders. Animal experiments have shown that oral TUDCA can enhance the insulin sensitivity in the liver and skeletal muscle of insulin resistant mice [31]. In a clinical study, it was found that the bile acid chelating agent can improve blood glucose levels in T2DM patients [32]. This mechanism potentially involves the intestinal excretion of bile acids upon their binding with bile acid chelating agents. This process effectively reduces the circulating bile acid content within the enterohepatic circulation. Subsequently, the decrease of the bile acid pool stimulates the liver to up-regulate bile acid synthesis to restore bile acid levels [33].

What’s interesting is that the profiles of BAs in patients with T2DM vary across different studies. Some studies have indicated that serum levels of primary and secondary BAs are significantly higher in T2DM patients compared to non-T2DM patients. Specifically, TCDCA, TDCA, HDCA, GDCA and GLCA show significant increases, while CA and TCA exhibit significant decreases [3]. However, a limitation of this study is the absence of stratified analysis based on the homeostatic model assessment of insulin resistance (HOMA-IR) value > 2.5. A similar study found no significant difference in total serum bile acid levels between the T2DM group and the normal group. Still, this study revealed that the levels of CA and DCA significantly increased after stratification based on HOMA-IR > 2.5 [34]. In summary, the composition of BAs in T2DM patients requires further analysis through additional clinical studies that consider relevant factors such as age, sex, geographic location, the use of bile acid drugs (e.g., metformin), and metabolic surgery [19, 35, 36].

### Bile acids and diabetes drugs (metformin and acarbose)

Metformin is one of the commonly used drugs in the treatment of T2DM, and its mechanism is complex. It has gained increasing attention for its role in reducing blood glucose and improving insulin sensitivity by affecting bile acid metabolism. Some studies have shown that in rat models of T2DM induced by Streptozocin (STZ) injection, oral metformin can inhibit weight gain, reduce CA synthesis, decrease the activity of CYP8B1, and ultimately ameliorate insulin resistance [37]. Simultaneously, it has been observed that metformin can enhance the expression of FXR and Musculoaponeurotic fibrosarcoma oncogene family protein G (MAFG) while inhibiting the expression of CYP8B1 in HepG2 cells. In clinical studies [35], metformin reduces blood glucose levels by increasing the secretion of Glucagon-like peptide 1 (GLP-1), a phenomenon confirmed in animal experiments. However, it's worth noting that these studies do not measure changes of BA concentrations. In addition, many studies have reported that metformin can inhibit the expression of the bile acid transporter BESP by activating cAMP-PKA and cAMP-AMPK pathways, although it does not appear to affect the expression of CYP7A1 [38–40]. This implies that metformin may influence not only bile acid synthesis, but also bile acid secretion and reuptake. However, it's essential to mention that the majority of studies on the effects of metformin on BSEP have primarily focused on non-alcoholic fatty liver disease, with limited reports available in diabetic rat models.

Acarbose is an α-glucosidase inhibitor and is commonly used in the treatment of T2DM. There have been relatively few studies on the effect of acarbose on BAs. In a multicenter, randomized controlled clinical trial [41], it was observed that the level of plasma secondary BAs (mainly DCA) and taurine-conjugated BAs decreased in patients with T2DM following treatment with acarbose. The mechanism behind this effect is that acarbose increases the relative abundance of Lactobacillus and Bifidobacterium while decreasing the relative abundance of Bacteroides.

We have summarized the hypoglycemic mechanisms and glucose outcomes of bile acid-related drugs, as shown in Table 1. In these studies, BAs (CDCA [42, 43], TUDCA [31, 44, 45], HCA [46], GUDCA [47]), FXR inhibitors (HS218[48], Gly-MCA [49, 50]), FXR agonists (Fexaramine [51, 52], GW4064 [53, 54]), FXR/TGR5 agonists (INT-767 [55]), TGR5 agonists (INT-777 [52], RO5527239 [52]), and clinical drugs (Colesevelam [56], Metformin [37, 57, 58], Acarbose [41], Obeticholic acid (OCA) [59–61]) all demonstrated hypoglycemic effects by activating different mechanisms. However, one study found that blood glucose increased in mice treated with GW4064 [62]. In fact, the increase in baseline blood glucose and the measurement of blood glucose levels 2 h postprandially may be influencing factors. Additionally, NGM282 is an analogue of fibroblast growth factor 19 (FGF19), but it has not been found to reduce blood glucose in clinical studies, indicating that FGF19 analogues may have a significant inhibitory effect on hepatic glucose synthesis, but may not directly affect glucose metabolism in other tissues or cells. This requires confirmation in future studies [63].

**Table 1:** Hypoglycemic mechanism and glucose outcome of bile acid-related drugs

Drugs	Animal experiment or clinical study	Change of bile acid	Main mechanism	Change in blood glucose
CA	Animal experiment(WKAH/HkmSlc rat)	–	Increase in Phylum Firmicutes and decrease in Bacteroides	Not mentioned [64]
CA	Animal experiment(WKAH rat)	Increase levels of TCA and DCA	1.Promote G6PD activation and pentose phosphate pathway 2.Increase in Phylum Firmicutes and decrease in Bacteroides	Not mentioned [65]
CDCA	Cell experiment(HIECs cell and IEC-6 cell)	–	Activate MEK/ERK signaling pathway, increase GLUT2 expression and promote glucose absorption	Reduce [42]
CDCA	Animal experiment(C57BL/6 mice)	–	Activate AC-PKA-cAMP pathway and stimulate hepatic spexin expression	Not mentioned [43]
TUDCA	Animal experiment(Swiss mice)	–	Activate TGR5-PKA-CREB pathway to promote insulin secretion	Reduce [44]
TUDCA	Animal experiment(C57BL/6 mice)	–	1.Increases insulin secretion without altering insulin clearance 2.Improve endoplasmic reticulum stress	Reduce [31]
TUDCA	Animal experiment(C57BL/6 mice)	–	Activate TGR5-cAMP-PKA pathway to promote insulin secretion	Reduce [45]
TUDCA	Animal experiment(db/db (C57BLKS/J-LepRdb/LepRdb) mice)	–	Inhibit endoplasmic reticulum stress and reduce the level of ER stress-related proteins (GRP78 and CHOP)	Reduce [66]
HCA	Animal experiment and Cell experiment(Bama miniature pigs (Sus scrofa), C57BL/6 J mice, STC-1 cell and NCI-H716 cell)	–	Inhibit FXR transcriptional activity, activate TGR5 pathway, promote GLP-1 secretion and insulin secretion	Reduce [46]
GUDCA	Animal experiment (C57BL/Ksj-db/db mice)	Increase UDCA、LCA、TUDCA、GUDCA、TLCA、T-α-MCA	Activate TGR5 pathway, promote PGC1A secretion and stimulate tissue thermogenesis	Reduce [47]
HS218	Animal experiment (C57BL/6 mice)		Inhibit FXR target genes SHP and BSEP, Reduce the mRNA levels of G6Pase and PEPCK	Reduce [48]
Gly-MCA	Animal experiment (C57BL/6N mice)	–	1. Inhibit ileal FXR-neuramide axis but do not affect the liver 2. No effect on TGR5-GLP1 signaling pathway	Reduce [49]
Gly-MCA	Animal experiment (C57BL/6N mice)	–	Inhibit ileal FXR-neuramide axis and reducs endoplasmic reticulum stress in the liver 2.Decrease SHP, Fgf15 and IBAP mRNA levels and unchange mRNA levels of Asbt	Not mentioned [50]
Fexaramine	Animal experiment (C57BL/6 J mice)	1.Bile: increase T-β-MCA, decrease T-α-MCA and TDCA, but do not affect TCA 2.Ileum: decrease CA, increase LCA 3.Colon: increase DCA and LCA 4.Serum: increase UDCA and TLCA	1.Increase secretion of FGF15 and FGF21 and GLP1 2.Increase expression of FXR target genes SHP, Fgf15, Osta/bRNAs, but not Asbt mRNA 3.Inhibitie expression of PEPCK and G6pase mRNA	Reduce [51]
Fexaramine	Animal experiment (foz/foz (Alsm1 ^−/−^) and WT (Alms1 ^+/+^) mice)	No change in LCA	1.Activate intestinal FXR signaling pathway but do not affect the liver 2.Do not affect the TGR5 signaling pathway	Reduce [52]
GW4064	Animal experiment ( C57BL/6 mice)	–	Activate intestinal FXR signaling pathway and increase SHP and FGF15 expression	Raise [62]
GW4064	Animal experiment and Cell experiment (C57BL/6 J mice and HK-2 cell)	–	Activate FXR-SHP signaling pathway and inhibite expression of PEPCK	Reduce [53]
GW4064	Animal experiment (C57BL/6 J mice)	–	Alleviation of hepatic steatosis and islet cell hypertrophy and improvement of insulin resistance, possibly related to NO metabolism	Reduce [54]
Cilofexor	Animal experiment (Wistar rat)	–	Activate FXR signaling pathway and promote expression of SHP and FGF15	Not mentioned [67]
INT-767	Animal experiment (C57BL/6 J mice)	1.Serum: decrease TCA, T-α-MCA, T-β-MCA, TDCA, T-CDCA, T-HCDA and T-MCDA 2.Liver:Increase T-β-MCA and T-α-MCA but decrease TCA 3.Ileum: decrease TCA and increase T-β-MCA and T-α-MCA	1.Liver: inhibit CYP7A1/cyp8b1 synthesis by activating the FXR-SHP signaling pathway 2.Ileum: activate FXR-FGF15-SHP pathway 3.Increase in Phylum Firmicutes and decrease in Bacteroides	Reduce [55]
INT-777	Animal experiment (foz/foz (Alsm1 ^−/−^) and WT (Alms1 ^+/+^) mice)	–	Activate TGR5 signaling pathway and promote GLP-1 secretion without affecting FXR	Reduce [52]
RO5527239	Animal experiment (foz/foz (Alsm1 ^−/−^) and WT (Alms1 ^+/+^) mice)	–	Activate TGR5 signaling pathway and promote GLP-1 secretion without affecting FXR	Reduce [52]
Colesevelam	Animal experiment (Zucker diabetic rats)	–	Promote miR-96/182/183 expression and inhibit MED1 expression	Reduce [56]
Metformin	Animal experiment and Cell experiment (Wistar rat and HepG2 cell)	Decrease CA	1.HEPG2 cells: promote FXR and MAFG expression and inhibit CYP8B1 expression 2.Mice: promote FXR and MAFG expression and inhibit CYP8B1 and CYP7A1 expression	Reduce [37]
Metformin	Animal experiment (C57BL/6 J mice)	Increase TUDCA	1.Activate Nrf2/ARE signaling pathway to improve insulin resistance 2.Decrease in bifidobacteria and increase in A. muciniphia	Reduce [57]
Metformin	Animal experiment (C57BL/6 J mice)	Increase CA、CDCA and decrease DCA	1.Inhibition of duodenal, colonic and ileal glucose transporter protein 5 expression 2.Increase in Verrucomicrobia	Unchanged (decrease but not statistically significant) [58]
Acarbose	Clinical study	Decrease DCA, taurine-conjugated BAs	Increase in lactobacillus and Bifidobacterium, decrease in Bacteroides	Reduce [41]
OCA	Animal experiment (db/db diabetic and obese mice)	–	Activate FXR-NrF2 signaling pathway and promote the expression of antioxidant enzymes SOD1, SOD2, CAT, GCLC and GPx	Reduce [59]
OCA	Clinical study	Decrease CA and TCA	1.Activate FXR signaling pathway to inhibit bile acid synthesis 2.Increase in Phylum Firmicutes	Not mentioned [60]
OCA and UDCA	Animal experiment (C57BL/6 J mice)	UDCA: Increase T-βMCA and TUDCA	UDCA: Activate TGR5 signaling pathway instead of FXR signaling pathway UDCA and OCA: 1.increase GLP-1 and FGF15 expression 2.decrease in Bacteroides	Reduce [61]
NGM282	Clinical study	–	Reduce HOMA-IR and suppress C4 levels	Unchange [63]

### Bile acids and bariatric metabolic surgery

Bariatric metabolic surgery can also achieve the effect of treating T2DM by changing the composition of BAs and improving insulin resistance [68–71]. However, there is controversy regarding changes in bile acid concentration after metabolic surgery. Some studies have found that the levels of CA, CDCA, TCA and total BAs decreased significantly 3 months after sleeve gastrectomy (SG), and the concentrations of CA and TCA still decreased significantly 6 months after SG [72, 73]. Furthermore, total BAs remained unchanged 6 months after SG, while primary BAs, including glycine and taurine-conjugated BAs, decreased [74]. In one study, serum C4 levels (a marker of bile acid synthesis) decreased from 23.4 ± 21.1 ng/mL at baseline to 4.9 ± 8.2,8.7 ± 12.1,13.8 ± 12.9 and 18.8 ± 16.8 ng/mL at 1,3,6 and 12 months after SG, suggesting a reduction in bile acid synthesis post-SG [75]. However, other studies found that the total serum BAs concentration increased more than threefold one year after Roux-en-Y gastric bypass (RYGB) [76]. Many studies suggested that the reason may be related to the increase of Fibroblast growth factor 19 (FGF19) [8, 77]. We believe that the reason for this contradiction lies in the duration of observation. A plausible explanation is that bile acid concentration decreases in the short term (1–3 months) after weight loss, but increases in the long term (1–5 years) after surgery. A second explanation could be the length of the intestinal loop, as different bariatric surgeries will impact pathway of bile acid entering the intestines, affecting bile acid recovery [78]. A third explanation may be that variations in diets, gender and ethnic backgrounds can also lead to diverse changes in BAs following bariatric surgery. Eastern patients tend to have higher initial TBA levels than Western patients, and a high-fat diet decreases primary bile acid synthesis [69, 76]. Additionally, male T2DM patients exhibit decreased TCA levels, while female patients experience reduced CA and TCA levels [3]. These factors can undermine the efficacy of weight loss and need to be taken into account. Furthermore, a patient's history of cholecystectomy may also affect bile acid reentry [79, 80].

### Bile acid and intestinal flora

As mentioned earlier, the intestinal flora mediates secondary bile acid synthesis. The most typical manifestation is that human circulating serum BAs are composed of CA, CDCA and DCA, whereas fecal BAs consist of DCA and LCA [81]. Interestingly, bile acid can also affect the composition of the intestinal flora. For example, in patients with T2DM, the relative abundance of Firmicutes decreased, and the relative abundance of Bacteroides increased [82]. Conversely, in rats fed with CA, the relative abundance of Streptomyces increased, while the relative abundance of Bacteroides and actinomycetes decreased [64]. This suggests that BAs may affect glucose metabolism by altering the composition of the intestinal flora. However, the mechanism by which the intestinal flora treats T2DM by influencing bile acid metabolism is still unknown and may be related to FXR and TGR5 [82, 83]. It has been found that acarbose can induce an increase in CDCA, enhance FXR activity, and simultaneously alter intestinal flora. However, their interactions are not certain, so it’s unclear while changes in the intestinal flora affect FXR activity [41]. Similarly, in animal experiments, it was observed that the intestinal flora after RYGB affected FXR and TGR5 by promoting the production of taurine-conjugated Bas [84]. A notable aspect of this study is the use of FXR inhibitor glycine-β-muricholic acid (Gly-MCA) to block intestinal FXR signals, which led to a reduction in the beneficial effects of the intestinal flora on glucose homeostasis. Simultaneously, it was noted that the intestinal flora had no significant impact on glucose tolerance and systemic insulin resistance in TGR5^−/−^ mice. However, it's worth mentioning that this study used obese rat models induced by a high-fat diet rather than the STZ-induced diabetic rat model, and the rats' blood glucose levels did not meet the criteria for T2DM.

The interaction between BAs and the intestinal flora plays a significant role in various physiological processes. BAs have been found to modulate blood glucose levels through multiple mechanisms, including alterations in intestinal pH, changes in the composition of the intestinal flora, and their impact on bacterial metabolites such as short-chain fatty acids and lipopolysaccharides [55, 61, 65]. On the other hand, the intestinal flora can influence bile acid metabolism by affecting the activity of enzymes such as BSH and 7-α-dehydroxylase, as well as by regulating bile acid transporters and reabsorption [16, 22]. While current research has primarily focused on animal experiments to investigate the changes in the intestinal flora following treatment with drugs that affect bile acid metabolism (such as FXR inhibitors, CA, OCA, etc.), there is a need for more clinical studies due to the inherent differences in dietary habits and genetic factors between humans and mice. Further research is necessary to understand the effects of BAs on the intestinal flora in patients with T2DM and elucidate the underlying mechanisms. Additionally, it is important to acknowledge that comparing results from different methods of sequencing the intestinal flora (such as high-throughput sequencing, metabolomics, and metagenomics) is scientifically unsound. Therefore, future studies should compare the strengths and limitations of these detection methods to identify an appropriate approach for studying bile acid metabolism.

## Hypoglycemic mechanism of bile acid

The hypoglycemic mechanism of bile acid is complex. Currently, the research is primarily focused on changes in bile acid composition and the effects of the bile acid signaling pathways on blood glucose. The pathways involved include the BA-FXR-SHP pathway [48, 51, 53], BA-FXR-FGFR15/19 pathway [77, 85, 86], BA-TGR5-GLP-1 pathway [7, 87, 88] and BA-TGR5-cAMP pathway [43, 44, 52]. Through these various pathways, the ultimate outcome is a modification in bile acid composition, a reductionin in the ratio of 12 α-hydroxylated BAs to non-12 α-hydroxylated BAs, an improvement in insulin resistance, and a decrease in liver gluconeogenesis and insulin sensitivity in adipose tissue. This is achieved by promoting insulin secretion and increasing energy consumption.

### FXR and bile acid

**Fig. 3:**
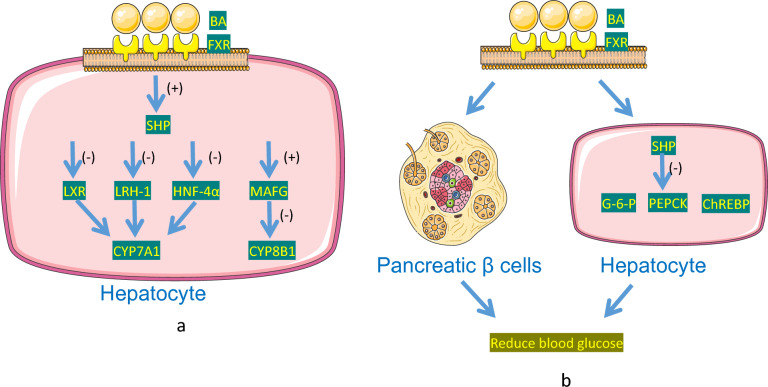
**a** dynamic balance of BAs. Intestinal and hepatic FXR work synergistically to regulate BAs, reducing their synthesis and promoting excretion. Activation of hepatic FXR leads to an upregulation of SHP, which in turn inhibits various transcription factors, including LXR, LRH-1, and HNF-4α, thereby impacting the activities of enzymes such as CYP7A1 and CYP8B1 involved in BA synthesis. Additionally, MAFG, influenced by FXR, may affect the CDCA: CA ratio, thereby influencing the hydrophobicity of BAs without altering the overall BA pool size. **b** BAs regulate blood glucose levels through FXR. BAs activate FXR within pancreatic β cells, which in turn promotes the release of insulin while inhibiting the release of glucagon and the production of glucose in the liver. SHP competes with HNF-4α, thereby suppressing the expression of genes related to gluconeogenesis. FXR’s direct interaction with ChREBP helps maintain glucose balance by reducing glycolysis and stimulating the storage of glycogen following a meal

The nuclear receptor FXR is a member of the ligand-activated nuclear receptors superfamily of transcription factors. In humans, FXRa is highly expressed in the adrenal gland and liver, while FXRb is highly expressed in the small intestine, large intestine and kidney [89]. Different BAs have varying effects on the activation of FXR, with the order of activation being CDCA > DCA > LCA > CA [13]. FXR plays a pivotal role in regulating glucose metabolism, impacting not only liver bile acid metabolism but also BA secretion and intestinal absorption. As the concentration of liver BAs increases due to intestinal reabsorption, intestinal and liver FXR work together to establish a dynamic balance in BAs by reducing bile acid synthesis and promoting bile acid excretion [90]. In the liver, the activation of FXR by BAs upregulates the expression of genes encoding the inhibitory nuclear receptor small heterodimer partner (SHP), particularly, with an increase in the bile acid pool size. SHP inhibits the activation of several transcription factors, including liver X receptor (LXR), liver receptor homologue-1 (LRH-1) and hepatic nuclear factor-4α (HNF-4α). This subsequently activates CYP7A1 in humans, inhibiting the initial step of cholesterol catabolism [11]. LXR stimulates bile acid synthesis by activating CYP7A1 transcription, but its effects are overridden in the presence of SHP [91]. Moreover, FXR activated by bile acids can increase the levels of MAFG, which subsequently inhibits CYP8B1 in mice. However, MAFG has no effect on CYP7A1. This suggests that MAFG may regulate the ratio of CDCA to CA, thereby controlling bile acid hydrophobicity (by inhibiting CA synthesis without affecting CDCA synthesis), but not the overallbile acid pool size [2, 13]. (Fig. 3a).

BAs promote insulin release, inhibit glucagon release and reduce glucose output from the liver by activating FXR in pancreatic β cells [46, 55, 92]. SHP competes with HNF-4 for binding, thus inhibiting the expression of various genes involved in gluconeogenesis, glucose transport and glycolysis. These genes include glucose-6-phosphatase (G6Pase) and phosphoenolpyruvate carboxylate kinase (PEPCK), which play essential roles in inhibiting liver gluconeogenesis, in particular, is the rate-limiting enzyme of hepatic gluconeogenesis. This FXR-mediated inhibition of key genes involved in glucose production leads to improved liver glucose utilization and uptake [48, 91, 93]. However, it should be noted that some data have shown that BAs may upregulate PEPCK through FXR [13, 18]. Furthermore, FXR plays a critical role in maintaining the dynamic balance of glucose through its direct interaction with the carbohydrate response element binding protein (ChREBP). ChREBP is an important transcription factor for glycolytic genes [19, 94]. In the postprandial state, activated FXR reduces glycolysis, promotes glycogen storage and inhibits de novo fat formation by repressing the expression of L-type pyruvate kinase. (Fig. 3b).

It has been observed that in intestinal-specific knockout mice (FXRΔIN), treatments such as Gastric Bypass with Ileal Interposition (GB-IL) or GS3672 [88, 95] did not result in altered blood glucose levels. This phenomenon may be attributed to the inability to activate the intestinal FXR signaling pathway, which does not alter GLP-1 secretion. However, a separate study indicated that, in comparison to FXR−/− mice, liver-specific knockout mice (FXRΔL) and intestinal-specific knockout mice still exhibit metabolic effects following VSG [96]. This study revealed that in FXR^ΔL^ and FXR^ΔIN^ mice, body weight decreased and blood glucose levels reduced after VSG, whereas these parameters remained unchanged in FXR^−/−^ mice. This findings suggests that liver and intestinal FXR knockout alone are insufficient to eliminate the beneficial effects of VSG. Moreover, this study found that the reduction in intestinal BA levels was not solely due to FXR activation in the intestine and liver. After VSG, Lactobacillus and Firmicutes were significantly increased in FXR^ΔL^ and FXR^ΔIN^ mice but remained unchanged in FXR-/- mice. Since Lactobacillus carries BSH and Firmicutes carries 7-α-dehydroxylase, this leads to a decrease in taurine-conjugated BAs in FXR^ΔL^ and FXR^ΔIN^ mice after VSG. These observations suggests that there may be more alternative pathways involved in BA metabolism after VSG, such as increased intestinal permeability and alterations in intestinal flora. These changes contribute to a decrease in BA levels.

In addition, some studies have found variations in BA changes across different regions when using the intestinal-specific FXR receptor agonist Fexaramine [51]. In general, this agonist increases the secretion of FGF15 and GLP-1, promoting insulin secretion and improving glucose tolerance by activating FXR signal pathway. On the contrary, the use of an intestinal-specific FXR receptor inhibitor, Gly-MCA, reduces intestinal ceramide by diminishing intestinal FXR signaling and inhibiting genes related to ceramide synthesis. This reduction in ceramide subsequently decreases hepatic endoplasmic reticulum (ER) stress and the production of proinflammatory cytokines, leading to improved blood glucose levels and insulin resistance [49]. Interestingly, these two drugs with completely opposite mechanisms yield similar results. However, this phenomenon actually reflects the diverse functions of intestinal FXR. It emphasizes the importance of considering not only functional alterations in FXR, but also potential changes in other mechanisms when investigating the relationship between BAs and FXR. Furthermore, it is crucial to determine whether FXR activation or inactivation varies among different tissues. Previous research has predominantly focused on mouse livers or primary hepatocytes, with limited exploration in other organs such as the intestine and kidney. Expanding the scope of investigation to encompass various tissues will provide a more comprehensive understanding of FXR’s roles.

### TGR5 and bile acid

TGR5 is a member of the G-protein-coupled receptor superfamily and is expressed in various tissues, including pancreas β Cells, endocrine cells in the small intestine, thyroid, brown adipose tissue, cardiomyocytes, and macrophages [11, 13]. It is not expressed in hepatocytes, but is instead located in sinusoidal endothelial cells [89]. TGR5 can be activated by a variety of BAs. Different BAs have varying abilities to activate TGR5 receptors, with LCA being the most potent, followed by DCA, CDCA, and CA [97]. Some studies have found that INT-767 can stimulate the secretion of glucagon-like peptide-1(GLP-1) in TGR5^−/−^mice, but not in FXR^−/−^mice, indicating that FXR is necessary for GLP-1 secretion. Subsequently, a FXR response element was discovered on the promoter of the TGR5 gene, suggesting that FXR is upstream of TGR5 [88, 98]. However, in the latest study, only CA7S (a sulfated metabolite of bile acid) was found to be increased in bile acid content in cecal contents after SG. Different concentrations of CA7S, however, were unable to activate endogenous FXR in human intestinal Caco-2 cells, suggesting that CA7S may induce TGR5 expression through an independent FXR mechanism [99].

**Fig. 4:**
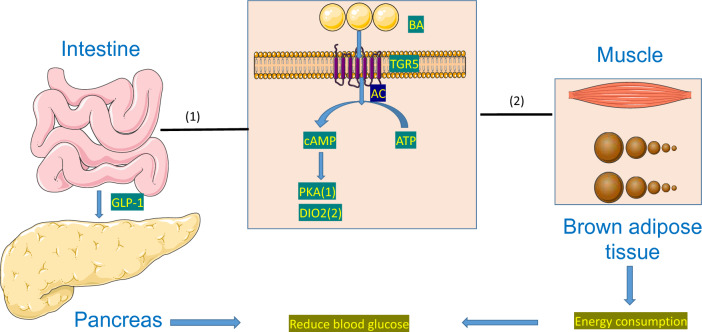
TGR5 and Bas. (1) Activation of TGR5 by BAs induces the expression of GLP-1, promoting insulin secretion through the PKA-CREB pathway. In the intestine, L cells activate TGR5, leading to increased GLP-1 secretion, regulation of glucose-dependent insulin and glucagon secretion, and the induction of satiety. (2) BAs regulate metabolism by enhancing thermogenesis and energy consumption. TGR5 activates thyroid hormones through DIO2, converting inactive T4 into its active form, T3, and increasing PGC1-α for local energy expenditure

TGR5 has been shown to induce the expression of GLP-1,thereby promoting insulin secretion [46, 61, 100]. With the help of its cofactors α, β and γ, TGR5 activates the protein kinase A (PKA) signaling pathway by stimulating adenylate cyclase, resulting in a rapid increase in intracellular cyclic adenosine monophosphate (cAMP) production. Then, the PKA pathway leads to the phosphorylation of the cAMP-response element-binding protein (CREB) and induces the expression of target genes [43, 44, 52]. In the intestine, endocrine L cells activate TGR5 to increase GLP-1 secretion.GLP-1 promotes insulin secretion by islet β cells and reduces glucagon secretion by islet alpha cells in a glucose-dependent manner [79, 101]. In addition to its glucose-dependent insulin-promoting effect, GLP-1 also shares characteristics with glucagon and induces a feeling of satiety [27, 102] (Fig. 4).

In addition, BAs regulate metabolic processes by regulating thermogenesis and increasing energy consumption. One explanation is that TGR5 activates thyroid hormones in brown adipose tissue and muscle cells by stimulating type II iodothyronine deiodinase (DIO2) [103]. The inactive thyroid hormone T4 is ultimately transformed into its active form T3, through the activation of the BA-TGR5-cAMP-DIO2 signaling pathway. This, in turn, increases peroxisome proliferator-activated receptor-gamma coactivator-1α (PGC1-α), stimulating localized energy consumption [27, 47, 104]. However, some studies have found that the triiodothyronine levels in mice after RYGB did not differ from those before surgery [103]. Another possible explanation currently under investigation is that more BAs arrives in the colon after metabolic surgery. This activation prompts intestinal endocrine cells to activate the BA-TGR5-GLP-1 axis, leading to increased GLP-1 release, which in turn facilitates muscle glucose absorption [87, 97, 105]. (Fig. 4).

In animal experiments, GW4064, an FXR agonist, have been shown to reduce blood glucose levels and improve insulin resistance by activating FXR and promoting GLP-1 secretion [54, 106]. Similarly, TGR5 agonists have exhibited similar effects [52]. However, in a rat intestinal perfusion model [107], whether through intracavitary perfusion or vascular perfusion, FXR agonists were unable to stimulate GLP-1 secretion, contrary to previous findings. Simultaneously, the TGR5 agonist RO6272296 had no effect on glucagon and insulin secretion in vitro pancreatic perfusion. This may appear inconsistent with previous studies, but it was later discovered that TGR5 was not expressed in neither islet α cells and β cells, which could explain this phenomenon. Despite these conflicting experimental results, both TGR5 and FXR agonists are generally considered beneficial for glucose metabolism.

We have summarized the primary characteristics, hypoglycemic mechanisms, and glucose outcomes of FXR and TGR5 knockout models, as shown in Table 2. When the same site in FXR knockout mice is subjected to different treatment measures, varying results are observed. Similarly, interventions of the same treatment yield diverse outcomes in different parts of the body. The potential reasons for these contradictions are as follows. Firstly, there is a variation in tissue specificity. Differences in the expression levels and regulatory mechanisms of FXR in different tissues may lead to distinct responses to the same treatment. Secondly, the complex nature of signal pathway mechanism plays a role. As a nuclear receptor, FXR participates in intricate signal pathways and transcriptional regulatory networks, resulting in potential cross-effects with other pathways and molecular interactions that give rise to different outcomes. Thirdly, discrepancies in experimental design and methods: differences in experimental design, interventions and evaluation criteria may also contribute to inconsistent results. For instance, variations in intervention dose, timing and experimental techniques can impact the results. In future research, a comprehensive examination of FXR's functions and regulatory mechanisms across various tissues and cell types will be crucial for a more precise understanding of its role in glucose metabolism. Additionally, it is essential to consider potential interactions. Exploring how FXR interacts with other signaling pathways and molecules is necessary to uncover the intricate regulatory networks that may help elucidate the currently contradictory findings.

**Table 2:** Main Performance, hypoglycemic mechanism and glucose outcome of FXR and TGR5 knockout models

FXR/TGR5	Animal models	Gene knockout targets	Intervention measures	Main Performance	Main mechanism	Change in blood glucose
FXR–/–	C57BL/6 J Mice	body	GW4064	GW4064 do not raise blood glucose, while 8-CPT-cAMP (PKA activator) raise blood glucose	In the presence of glucagon, GW4064 promote gluconeogenesis through the CCG-PKA signaling pathway by promoting Fbp1, Pck1, and G6pc expression In the presence of cAMP analogs, it inhibits gluconeogenesis through the SHP / Nr0b2 signaling pathway	Raise [94]
FXR–/–	C57BL/6JMice	body	VSG	Body weight and blood glucose are not improved after VSG	Reduce expression of FXR transcriptional target genes 2. no change in intestinal Bacteroides 3. BA in the liver remained unchanged, while CA, TCA and GCA in the intestine decreased 4.TCA supplementation reverses the metabolic benefits of VSG	Unchange [96]
FXR–/–	Not mentioned	body	–	Raise blood glucose and blood lipids Aggravate diabetic cardiomyopathy, diabetic myocardial fibrosis	Increase expression of lipid synthesis and lipid transport genes in diabetic cardiomyocytes (SCD-1, SREBP-1c, FAS, ACC, LOX-1, LDLR)	Raise [108]
FXR–/–	Not mentioned	body	BD	FXR-/- mice are resistant to HFD-induced obesity	1.BD significantly increase the expression of PGC-1ß in the liver of WT mice, but BD faile to promote FXR-/- mice 2.Increase in Proteobacteria, decrease in Bacteroides	Rasie [109]
FXR–/–	C57BL/6 J mice	body	RYGB	FXR-/- mice slow weight gain and improve glucose homeostasis	The GLP-1 antagonist Ex-9 attenuate the postoperative hypoglycemic effect of RYGB in the control group but do not alter the FXR-/-group, suggesting an association with the FXR-GLP-1 axis	Reduce [110]
FXR–/–	C57BL/6 mice	intestine	GB-IL	No change in weight after GB-IL, no improvement in glucose tolerance	Failure of BAs to activate FXR pathway and unaltered GLP-1 levels	Unchange [88]
FXR–/–	Not mentioned	intestine	GS3972	1. ileal expression of Fgf15 was significantly reduced, Cyp7a1 expression was significantly higher in the liver, and Cyp8b1 was not affected 2. Long-term HFD resulted in impaired glucose metabolism and reduced insulin sensitivity in WT mice and FXR-/- mice	1.Liver: GS3972 treatment significantly increase the expression of FXR target genes SHP in WT and FXR-KO mice, while Cyp7a1 and Cyp8b1 expression is significantly decreased 2.Ileum: Fgf15 and ileal bile acid binding protein (I-BABP) are expressed in the ileum of WT mice after GS3972 treatment, but not in FXR-KO mice	Unchange [95]
FXR–/–	C57BL/6 J Mice	intestine	VSG	Reduce body weight and improve insulin sensitivity	intestine:decreased levels of taurine-conjugated BAs 2.Increase in Firmicutes and Lactobacillus	Reduce [96]
FXR–/–	C57BL/6 J Mice	Liver	VSG	Reduce body weight and improve insulin sensitivity	1.intestine:decreased levels of taurine-conjugated BAsBAs 2.Increase in Firmicutes and Lactobacillus	Reduce [96]
FXR–/–	C57BL/6 J Mice	Adipocyte	–	Do not improve HFD-induced obesity but improve insulin sensitivity	Promote AKT phosphorylation, promote GSTA4 expression and reduce oxidative stress	Reduce [111]
TGR5–/–	C57BL/6 J Mice	body	VSG	Decrease in body weight, no change in energy expenditure, no change in plasma insulin concentration	Cyp8b1 expression is inhibited and the ratio of 12α-OH:non-12α-OH BAs is increased	Unchange [112]
TGR5–/–	C57BL/6 J Mice	body	HDCA	1. insulin secretion is higher and there is no effect on blood glucose 2. ω-MCA and HDCA levels in the portal vein are increased only in the WT mice. group, but not in the TGR5- KO mice group,	HDCA promotes insulin secretion and regulates blood glucose by activating the TGR5-GLP-1 signaling pathway	Unchange [113]
TGR5–/–	C57BL/6 Mice	body	GB-IL	GB-IL postoperative weight loss and improved glucose tolerance	Associated with activation of FXR by BAs and promotion of GLP-1 secretion	Reduce [88]
TGR5 overexpression	C57BL/6 mice	skeletal muscle	TLCA	Levels of glucose 6-phosphate (G6P) and fructose 6-phosphate (F6P) are significantly reduced in Tg mice	Promote glycolysis, do not improve muscle insulin sensitivity, do not improve insulin resistance	Unchange [105]

### FGF15/19 and bile acid

**Fig. 5:**
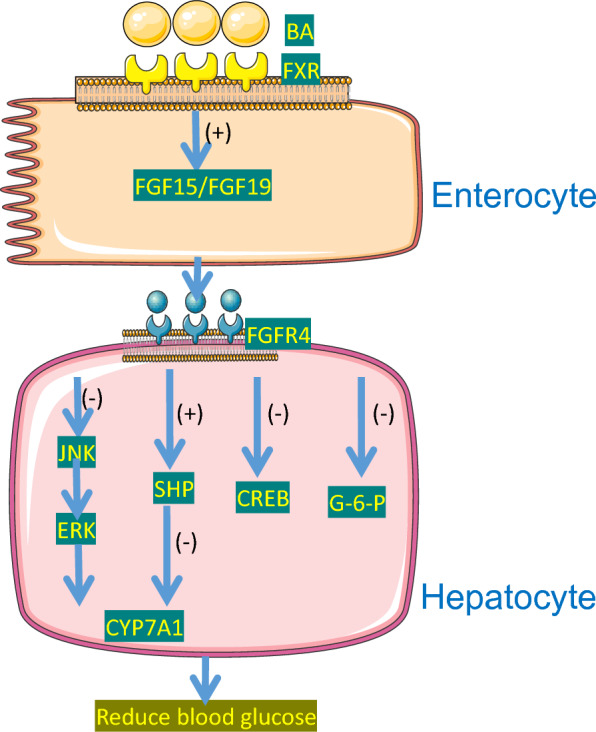
FGF15/19 and BAs. FGF19 inhibits BAs synthesis by binding to FGFR4, thus blocking CYP7A1 through two pathways: SHP-independent inhibition via JNK/ERK and SHP-dependent activation via KLB. FGF19 increases inhibits hepatic gluconeogenesis via CREB and downregulates glucose 6-phosphatase gene expression, while activating FGFR4/KLB promotes glycogen synthesis and inhibits hepatic gluconeogenesis and adipose tissue glucose disposal, contributing to its hypoglycemic effects

FGF19 is a member of the hormone-like FGF protein family, synthesized in the distal small intestine or ileum, gallbladder and brain in humans, while FGF15 is synthesized in mice [28, 114]. It has been reported that there is a strong positive correlation between postprandial total BAs and FGF19, possibly because BAs bind to FXR and stimulate FGF19 synthesis [100]. FGF19 is released from the distal small intestine, enters the portal vein circulation, and acts on the liver. It follows a circadian rhythm, with its peak occurring 90 to 120 min after the rise in serum bile acid levels following meals [115]. Fibroblast growth factor receptors (FGFRs) include FGFR1, FGFR2, FGFR3 and FGFR4. FGF19 inhibits CYP7A1 by binding to FGFR4, thereby inhibiting BAs synthesis [32, 98]. The first regulatory mechanism involves FGF19 binding to FGFR4 on the plasma membrane of hepatocytes to inhibit CYP7A1 transcription, mediated by c-Jun amino-terminal kinase/extracellular signal-regulated kinase (JNK/ERK). This is an effective SHP independent pathway to inhibit CYP7A1 [51, 55, 62]. The second regulatory mechanism is that FGF19, with the help of β-Klotho (KLB) in hepatocytes, directly activates SHP and inhibits CYP7A1 by binding to FGFR4 [116]. (Fig. 5).

Clinical studies have revealed a negative linear correlation between FGF19 levels and indicators of T2DM severity (including Hemoglobin A1c (HbA1c) and C-peptide levels) [115]. This correlation can be attributed to several factors. Firstly, the increase in FGF19 levels inhibits hepatic gluconeogenesis by suppressing the transcriptional activity of CREB, which is a key regulator of PGC-1α. Secondly, FGF19 demonstrates its hypoglycemic effects by down-regulating the expression of glucose 6-phosphatase gene [28]. Lastly, FGF19 activates FGFR4/ β-Klotho in the liver, promoting glycogen synthesis and inhibiting gluconeogenesis in the liver, as well as enhancing glucose disposal in adipose tissue [89, 104]. However, some studies have suggested that the reduction in circulating glucose levels and the improvement in glucose tolerance in obese mice due to FGF-19 are unrelated to insulin secretion. Instead, these effects are attributed to reduced intestinal glucose uptake in the digestive limbs lacking bile acids [7].

In addition, it has recently been considered that the role of FGFR1 in fat and glucose metabolism cannot be ignored. Within the pancreas, FGFR1 is primarily located in β-cells, and a reduction in FGFR1 signaling can lead to β-cell dysfunction. Studies have shown that FGFs-FGFRs binding also activates PI3K/Akt pathway, which mediates metabolism [116, 117].

### Other pathway mechanisms and bile acids

In addition to acting on FXR and TGR5, BAs can also regulate blood glucose and affect metabolism through pregnane X receptor, constitutive androstane receptor, vitamin D receptor and other receptors [118]. LCA activates pregnane X receptor and vitamin D receptor, binding to the BA response element-I sequence in the CYP7A1 promoter to inhibit the activity of CYP7A1 promoter [14]. In rodent hepatocytes, DCA activates epidermal growth factor receptor ERB1/ERB2 and insulin receptor through PI3K/AKT/GSK3 signal pathway to participate in the activation of glycogen synthase [13]. BAs can also perform many other biological functions through non-receptor interactions, involving JNK1/2, ERK1/2, Akt1/2 signal pathways, NO metabolism, and cationic channel activation.

Obesity-induced endoplasmic reticulum (ER) stress has been implicated in the development of insulin resistance and T2DM [66]. Additionally, an increase in BAs following weight loss may improve glucose regulation by mitigating ER stress in peripheral insulin-sensitive tissues. A study demonstrated that ER stress signals in the liver, adipose tissue, and pancreas decreased after undergoing ileal transposition surgery [119]. Another study showed that oral administration of TUDCA inhibited neointimal proliferation in mouse vascular smooth muscle cells and reduced ER stress in endothelial cells [73]. However, the understanding of the relationship between BAs and ER stress remains limited, and the underlying mechanisms are not fully elucidated. Existing research suggests that ER stress can lead to the upregulation of PERK, promoting islet β-cell apoptosis, inhibiting insulin secretion, and inducing insulin resistance [120, 121]. Conversely, BAs can suppress the expression of ER stress-related proteins, improve insulin resistance, and reduce blood glucose levels by modulating the JNK signaling pathway [31, 66]. Due to the current lack of relevant research, future investigations should focus on two aspects: firstly, evaluating the effects and mechanisms of different types of BAs on ER stress, which could provide valuable insights for the development of hypoglycemic drugs. Secondly, assessing whether there are variations in ER stress among different tissues (e.g., liver, skeletal muscle, adipocytes), as this could identify novel targets for the treatment of T2DM.

Impairment of bitter receptor function has been implicated in the increased prevalence of T2DM, highlighting the potential role of BAs, which are natural bitter substances, in glucose homeostasis. Activation of intestinal bitter taste receptors has been shown to induce GLP-1 secretion, facilitate weight loss, and improve glucose tolerance in rodent models [122]. Previous studies have suggested a possible connection between BAs and intestinal bitter taste receptors. Certain drugs, including KDT501 [123], berberine [124], and Cucurbitacin B [125], have been reported to activate bitter taste receptors and stimulate GLP-1 secretion. Recent research has further demonstrated the ability of LCA and TLCA to activate intestinal bitter taste receptors [126]. Notably, LCA has been found to activate Taste Receptor Type 2 Member 1 (TAS2R1) in humans at a concentration of 0.3 µM, while TLCA activates TAS2R1, TAS2R14, and TAS2R46 at the same concentration. In mice, TLCA activates TAS2108 at 1 µM, and TLCA activates TAS2144, whereas LCA activates TAS2105 at 3 µM. These observations suggest that human gut bitter taste receptors exhibit heightened sensitivity to BAs. However, the underlying mechanism of this activation remains elusive and warrants further investigation, including the exploration of potential involvement of the TGR5 pathway using TGR5 knockout mice. In summary, BAs, acting as natural bitter substances, have the capacity to activate receptors associated with bitterness perception. The impairment of bitter receptor function has been associated with an increased risk of T2DM.

## Conclusion

The relationship between bile acid metabolism and T2DM has attracted much attention. In recent years, the research in this field has made rapid progress, and the content involved is more and more extensive. This review mainly discusses the relationship between bile acid and T2DM, summarizes the latest research, analyzes the limitations of existing research and looks forward to the future development trend.

Current studies have shown that BAs are a group of natural compounds produced in the liver and excreted through the intestines. BAs regulate insulin secretion, liver glycogen synthesis and intestinal glucose absorption by activating nuclear receptors such as FXR, TGR5 and FGF15/19 signal transduction pathways, thus affecting glucose metabolism and energy balance. The relationship between BAs and T2DM is also supported in some related clinical studies. For example, data show that there is a significant association between T2DM and serum BAs levels. Bile acid replacement therapy has also been proved to be effective in the improvement of T2DM. We further discussed the molecular mechanisms and signal transduction pathways of bile acid metabolism and T2DM. Investigating the molecular mechanisms in T2DM pathogenesis enhance our comprehension of bile acids' influence on metabolic control. This includes alterations in intestinal flora composition, regulation of genes associated with bile acid synthesis and transport, and modulation of intestinal endocrine hormone secretion. These processes subsequently impact glucose metabolism.

Although the existing studies have preliminarily revealed the relationship between bile acid and T2DM, there are still some limitations. Firstly, due to the interaction between bile acid metabolism and T2DM, it is not clear whether the abnormal bile acid metabolism is the cause or result of T2DM. Secondly, bile acid metabolism is a complex mechanism involving multiple pathways. Current studies mainly focus on the effects of bile acid on FXR and TGR5, ignoring other potential pathways such as endoplasmic reticulum stress, bitter receptor, intestinal flora and so on. At the same time, most studies mainly explore the effects of bile acid on liver and intestinal tract, ignoring the heart, kidney and brain tissue. Thirdly, the experimental models of most studies are based on mice or small-scale people, this method is limited to superficial understanding, and there are certain restrictions on the generalization of conclusions. Fourthly, some studies often lack the concept of personalized treatment, ignoring the differences between different patients. Lastly, some of the experimental results are contradictory, such as in FXR knockout mice, the same site of gene knockout but the results are different. In addition, the change of bile acid concentration during weight loss metabolic surgery is also controversial.

In view of this, there are several potential approaches for future research on BAs and T2DM. Firstly, for the causal relationship between bile acid metabolism and T2DM, Genome-Wide Association Studies and long-term longitudinal studies with large samples can be considered. Secondly, further research on the mechanism can focus on the relationship between bile acid and intestinal flora, bitter receptor and endoplasmic reticulum stress, as well as the effects of bile acid on heart, kidney and brain tissue. Thirdly, to explore the differences in the efficacy of bile acid therapy among different populations, such as genetic variation or intestinal microflora, in order to develop a more individualized treatment plan. Fourthly, the potential side effects of bile acid therapy, such as gastrointestinal problems or abnormal liver function, need to be further studied and evaluated. Ensuring safety is one of the important factors to promote the development of this field. Fifthly, the development of new bile acid analogues or bile acid receptor agonists may have a greater hypoglycemic effect on patients. Lastly, strengthen the multi-disciplinary cooperation of bile acid metabolism and T2DM research, integrate the knowledge of genetics, metabolism, intestinal microbiology, systems biology and other disciplines, in order to deepen the overall understanding of its complex relationship.

## Data Availability

Not applicable.

## Abbreviations

T2DM: Type 2 diabetes mellitus
BA: Bile acid
FXR: Farnesoid X receptor
TGR5: G-protein-coupled bile acid receptor 5
CDCA: Chenodeoxy cholic acid
CA: Cholic acid
CYP7A1: Cholesterol 7 α-hydroxylase
C4: α-Hydroxy-4-cholesterol-3-one
CYP8B1: Cholesterol 12 α-hydroxylase
CYP27A1: Cytochrome P450 family 27 subfamily A member 1
BSEP: Bile salt export pump
MRP2: Multidrug resistance-associated protein 2
ASBT: Apical sodium-dependent bile acid transporter
MRP3: Multidrug resistance-associated protein 3
IABP: Ileal bile acid binding protein
OST α/OST β: Organic solute transporter α/β
NTCP: Sodium-dependent taurocholate cotransporting polypeptide
OATP: Organic anion transporter
DCA: Deoxycholic acid
LCA: Lithocholic acid
BSH: Bile salt hydrolase
UDCA: Ursodeoxycholic acid
TCA: Taurocholic acid
HOMA-IR: Homeostatic model assessment of insulin resistance
MAFG: Musculoaponeurotic fibrosarcoma oncogene family protein G
GLP-1: Glucagon-like peptide 1
AMPK: AMP-activated protein kinase
SG: Sleeve gastrectomy
FGF15/19: Fibroblast growth factor 15/19
RYGB: Roux-en-Y gastric bypass
Gly-MCA: Glycine-β-muricholic acid
SHP: Small heterodimer partner
LXR: Liver X receptor
LRH-1: Liver receptor homologue-1
HNF-4α: Hepatic nuclear factor-4α
G6Pase: Glucose-6-phosphatase
PEPCK: Phosphoenolpyruvate carboxylate kinase
ChREBP: Carbohydrate response element binding protein
FXRΔIN: Intestinal-specific FXR knockout mice
FXRΔL: Liver-specific FXR knockout mice
ER: Endoplasmic reticulum
PKA: Protein kinase A
cAMP: Cyclic adenosine monophosphate
DIO2: Type II iodothyronine deiodinase
PGC1-α: Peroxisome proliferator-activated receptor-gamma coactivator 1-α
FGFRs: Fibroblast growth factor receptors
JNK/ERK: C-Jun amino terminal kinase/extracellular signal regulated kinase
KLB: β-Klotho
PXR: Pregnane X receptor
CAR: Constitutive androstane receptor
VDR: Vitamin D receptor
TAS2R: Taste 2 receptor
